# Responsiveness of the Italian version of the Pediatric Quality of Life Multidimensional Fatigue Scale in adult inpatients with obesity

**DOI:** 10.1038/s41598-022-15261-z

**Published:** 2022-07-13

**Authors:** Matthew F. Smout, Gian Mauro Manzoni, Anna Guerrini-Usubini, Diana Caroli, Alessandra De Col, Gianluca Castelnuovo, Giada Pietrabissa, Enrico Molinari, Alessandro Sartorio

**Affiliations:** 1 Justice and Society, University of South Australia, Adelaide, SA Australia; 2 Faculty of Psychology, eCampus University, Via Isimbardi 10, 22060 Novedrate, Como Italy; 3 Clinical Psychology Research Laboratory, IRCCS, Istituto Auxologico Italiano, Milan, Italy; 4 Department of Psychology, Catholic University of Milan, Milan, Italy; 5 Experimental Laboratory for Auxo‑Endocrinological Research, IRCCS, Istituto Auxologico Italiano, Piancavallo-Verbania, Italy; 6 Division of Auxology and Metabolic Diseases, IRCCS, Istituto Auxologico Italiano, Piancavallo-Verbania, Italy

**Keywords:** Psychology, Endocrinology, Health care

## Abstract

This study aimed to evaluate the responsiveness of the Italian version of the Paediatric Quality of Life Inventory Multidimensional Fatigue Scale (PedsQL-MFS) to changes in BMI, fatigue and depressive symptoms in adult inpatients with obesity. 198 adults (81% female, mean age = 44.7 years) with obesity completed the PedsQL-MFS, the Fatigue Severity Scale (FFS) and the Centre for Epidemiologic Studies Depression Scale (CESD) before and after completing a 3-week body weight reduction program. Internal responsiveness was measured via paired t-tests, standardized mean response (SMR) and Glass’s delta (*d*). Changes in FFS, CESD and BMI were used as anchors to categorize participants as “improved”, “unchanged” or “deteriorated”. External Responsiveness was assessed by comparing mean post-intervention PedsQL-MFS scores across change groups, adjusting for pre-intervention PedsQL-MFS scores and in area-under-curve (AUC) analysis. PedsQL-MFS Total, Sleep/Rest Fatigue and Cognitive Fatigue scores demonstrated significant reductions in response to an established body weight reduction program. Post-intervention PedsQL-MFS scale scores were lower among those who had improved on the CESD and FSS than among those whose CESD and FSS scores had not significantly changed. There was no difference in PedsQL-MFS scale scores according to whether participants had reduced their BMI by at least 5%. AUC analyses indicated that change in PedsQL-MFS scores was somewhat more predictive of improvement in CESD than FSS scores. The Italian version of the PedsQL-MFS demonstrated both internal and external responsiveness. It appeared more sensitive to improvement than deterioration in fatigue symptoms and its sensitivity to deterioration in depressive symptoms and weight loss could not be evaluated in the present study as there was no reliable deterioration in CESD scores and weight loss was modest. Future studies should include a control group to assess the sensitivity of the PedsQL-MFS more thoroughly.

## Introduction

Obesity—defined as an excess of body fat^1^ is a significant risk factor for a plethora of physical, psychological, and social problems, all of which can heavily impact health, quality of life, and functioning. Consequently, obesity was recently defined as a disabling condition concerning physical, psychological, and social status^2^. Obesity contributes to the onset of comorbidities such as metabolic, musculoskeletal, or heart diseases^3^. Obesity is a well-established risk for developing medical conditions, including type II diabetes mellitus, osteoarthritis, chronic pain, heart failure, coronary heart disease, and hypertension^4^. Obesity also increases the risk for mental health problems, such as depression, anxiety, and eating disorders^5^. Physical disability related to obesity generally impairs daily living activities, indoor and outdoor mobility, housework, and occupational activities^6^ reducing quality of life. Obesity also negatively impacts sleep quality^7^; individuals with obesity often complain of excessive daytime sleepiness and fatigue^8–11^.

Individuals with obesity self-report higher levels of fatigue than people of normal weight^12^. Fatigue has been associated with higher BMI and waist circumference, and with less likelihood of getting sufficient physical activity^10^. Fatigue, in turn, negatively impacts health, quality of life, and daily functioning. Fatigue itself has been shown to be a strong predictor of poor job performance, job turnover, and incidence of physical illness^7^.

Although excessive daytime sleeping and fatigue have often been considered the same states, there are some differences between the two constructs. In particular, excessive daytime sleepiness has been defined as an excessive amount of sleep or a difficulty in maintaining wakefulness^13^, while fatigue was defined as a subjective perception of lack of energy, apathy and physical as well as psychological tiredness^14–16^. Both entail a subjective experience of feeling physically and psychologically tired, but while excessive daytime sleepiness is associated with increased sleeping propensity^17,18^, fatigue is not^19^.

Despite sleepiness and fatigue, which have been associated with obesity^10,20^, having a severe impact on individuals’ well-being, they remain understudied. However, some evidence suggests that obesity is associated with daytime sleepiness independently of sleep disturbances, such as sleep apneas or sleep disruption^21,22^. Other studies also revealed that determinants of sleepiness and fatigue were depression and metabolic factors such as obesity and diabetes. Finally, sleepiness and fatigue were also found to be related to insulin resistance^23^. Taken together, these findings have led Vgontzas and colleagues^19^ to refuse the hypothesis that sleep disturbances, such as sleep apneas and sleep disruption, were the primary factors involved in sleepiness and fatigue in the obese population, in favor of a specific role of obesity per se in determining sleepiness and fatigue. Specifically, the authors suggested that sleepiness is primarily related to metabolic factors, such as diabetes and insulin resistance, while fatigue is associated with psychological factors, such as depression.

Despite the well-recognized negative impact of sleepiness and fatigue in obesity, to the authors’ knowledge only one study assessing the efficacy of a residential treatment for obesity included fatigue (and not sleepiness) among the outcome variables^24^. In particular, Sartorio and colleagues’ study^24^ showed that a three-week multidisciplinary rehabilitation program for weight loss, including physical activity, effectively reduced weight, fatigue perception, and lower limb anaerobic power output in a sample of 200 inpatients with obesity.

In Sartorio et al.’s study^24^, fatigue was measured by the Fatigue Severity Scale (FSS), where the scores were shown to be valid and reliable, and responsive to an inpatient body weight reduction program in a sample of 220 individuals with obesity^25^.

Another tool for assessing fatigue is the Pediatric Quality of Life Inventory Multidimensional Fatigue Scale (PedsQL MFS)^11^. It covers three domains of the fatigue construct: general fatigue, sleep/rest fatigue, and cognitive fatigue. Although originally designed for pediatric patients, because many pediatric patients stay with their health care providers into adulthood, the validity of the PedsQL MFS in adults was explored and established^26^. The Italian version of the PedsQL MFS for adults over 26 years old^16^ has shown to be feasible and produce valid and reliable measures of fatigue. However, information about its internal and external responsiveness is lacking.

Establishing the responsiveness of a measure, also known as its sensitivity to change, involves demonstrating that scores on the measure change in response to known meaningful clinical changes as indicated by some other indicator. Internal responsiveness is demonstrated when a measure changes in a group exposed to an intervention known to be effective. External responsiveness is demonstrated when a new measure changes over time in a group selected because it has already changed according to an established outcome measure^27,28^. An unresponsive measure is of no value in monitoring clinical status of patients. Conversely, the more sensitive a measure is to change, the lower the risk of false negative conclusions about response to interventions, and the lower the sample size requirements and associated costs involved in conducting research using that outcome measure^29^. The current study aimed thus to assess both the internal and external responsiveness of the Italian version of the PedsQL MFS following a 3-week multidisciplinary rehabilitation program for inpatients with obesity. The external responsiveness measures chosen in this study were the Fatigue Severity Scale and the Community Epidemiologic Studies of Depression (CESD) scale because their Italian versions have been previously validated for use in our inpatient program^25^ and are now routinely used in service evaluation.

## Methods

### Participants

One hundred ninety-eight adults with obesity (160 females and 38 males, age *rng* = 18–76, *M* = 44.4, *SD* = 6.1), hospitalized at the Division of Metabolic Diseases, Istituto Auxologico Italiano, IRCCS, Piancavallo (VB), Italy, for a 3-week multidisciplinary body weight reduction program, participated in the study after giving their written informed consent.

The inclusion criteria were individuals of both sexes, older than 18 years, having a BMI > 35 kg/m^2^.

The study protocol was approved by the Ethical Committee of the Istituto Auxologico Italiano (research project code: 01C403, acronym: QOLFATIGUEOB) and all research was performed in accordance with the Declaration of Helsinki.

Informed consent was obtained from all participants, who were administered all measures listed below on two occasions: on the first day of hospitalization and then again, prior to discharge.

### Measures

#### Pediatric Quality of Life—Multidimensional Fatigue Scale (PedsQL-MFS)

The PedsQL-MFS^30^ is an 18-item measured comprised of three subscales: Sleep/Rest Fatigue (e.g.*,* “I sleep a lot”); Cognitive Fatigue (“It is hard for me to keep my attention on things”) and General Fatigue (“I feel physically weak (not strong)”). The frequency with which each symptom is a problem is rated from 0 “almost never” to 4 “almost always”. In a previous study involving the same sample^16^, the Italian version of the PedsQL-MFS subscales showed strong internal consistency, generally strong correlations with depression symptoms, and the Total and General Fatigue scales displayed moderate correlations with the Fatigue Severity Scale, but the subscales showed significant floor effects. Although Manzoni and colleagues^16^ found a modifed version of the original 3-factor structure best fit the data, the subscales in the present study were calculated using the orignal structure^30^. In the present study, internal consistency values were: General Fatigue α = 0.92; Sleep/Rest Fatigue α = 0.74; Cognitive Fatigue α = 0.92; Total α = 0.93. Percentages of floor effects as indicated by a raw score of 0 + minimal detectable change (MDC) and ceiling effects defined as a maximum raw score—MDC were: General Fatigue 5.6% and 2%; Sleep/Rest Fatigue 5.6% and 0.5%; Cognitive Fatigue 12.1% and 0.5%; Total 2.5% and 0%. Thus, floor and ceiling effects were within acceptable limits (< 15%) in the present study.

#### Fatigue Severity Scale (FSS)

The Fatigue Severity Scale^31^ consists of 9 statements about the impact of fatigue on functioning (e.g., “My motivation is lower when I am fatigued”) each rated on a scale from 1 “strongly disagree” to 7 “strongly agree”. In a sample of similar inpatients with obesity, the Italian version displayed high internal consistency, convergent validity via a strong correlation with the POMS-Fatigue scale score, discriminant validity via a negative correlation with the POMS-Vigor score, and responsivity to medium effect size changes in fatigue^25^. In the present study, the FSS displayed strong internal consistency (α = 0.94). Floor and ceiling effects were well within acceptable limits (4% and 1% respectively).

#### Community Epidemiologic Studies Depression Scale (CES-D)

The CES-D^32^ is a 20-item questionnaire measuring somatic, negative affect and anhedonic symptoms of depression (e.g., “I felt lonely”) with 4 reverse-worded items (e.g., “I felt hopeful about the future”)^33^. The frequency of each symptom is rated from 0 “rarely or none of the time” to 3 “most or all of the time”. The Italian version has been found to have good internal consistency and to discriminate between individuals with and without major depressive disorder^34^. The internal consistency in this study was α = 0.88. There were 0% floor or ceiling effects.

### Body weight reduction program (BWRP)

All participants underwent the 3-week BWRP including a Mediterranean personalized diet with an energy content obtained by subtracting ∼30% from total energy expenditure, which is obtained by multiplying the mREE by the physical activity level during the BWRP.

Diet composition was: 18–20% proteins, 50–55% carbohydrates (< 15% simple sugar), 27–30% lipids (< 8% saturated fat), and ∼30 g of fibers. Foods which the patient declared they were allergic to were removed from the menu. Five daily portions of fruits and vegetables were mandatory and a fluid intake of at least 1.5 L/day was recommended.

During the BWRP all participants had educational lessons on nutrition, consisting of lectures, demonstrations and group discussions with and without a supervisor, which took place every day throughout the whole BWRP period.

The BWRP included also a physical activity program consisting of 5 days per week training including (i) 1-h dynamic aerobic standing and floor exercises with arms and legs, at moderate intensity and under the guidance of a therapist; (ii) either 20–30 min cycloergometer exercise at 60 W, or 3–4 km outdoor walking on flat terrain, according to individual capabilities and clinical status. In addition, participants had 1 h/day of aerobic free activities at the institution on Saturday and Sunday.

Each participant also received psychological sessions led by a clinical psychologist 2–3 times per week, which were based on cognitive-behavioral strategies with individual or group sessions.

### Data analysis

The Italian PedsQL-MFS internal responsiveness was evaluated via paired t-tests on pre- and post-intervention mean scores for the total and each subscale. Within-group effect sizes for Glass’s delta (mean change/Pre *SD*) and the standardized mean response (SMR, Mean change/*SD*_CHANGE_) were calculated.

External responsiveness was evaluated via two methods: (1) by creating “improved”, “unchanged” and “deteriorated” groups on the external measures and comparing differences in PedsQL-MFS scores between baseline and post-intervention between these groups; and (2) receiver operating characteristics (ROC) analysis of the accuracy of PedsQL-MFS mean change scores to categorize clients as “improved” or “not improved” (i.e., unchanged or deteriorated) on each external measure. For the FSS, Impellizeri and colleagues^25^ found a minimal detectable change value of 1.2 for the Italian version which was greater than other estimates of minimally important differences^35^; participants whose FSS scores decreased by 1.2 points or more were classified as “improved”, those whose FSS scores increased by 1.2 points or more were classified “deteriorated” and the rest were “unchanged”. For the CES-D, a reliable change index^36^ was calculated for the sample assuming the test–retest reliability would approximate the validation study 4-week value—*r* = 0.67^32^. Accordingly, participants who achieved decreases in CES-D total scores of 13.53 (reliable change index) or more were classified “improved”, those whose CES-D scores increased by 13.53 were classified “deteriorated” and the remainder were “unchanged”. For BMI, a reduction of 5% was considered clinically significant^37^ so those whose BMI had reduced by ≥ 5% were classified “improved”, those whose BMI increased by ≥ 5% “deteriorated” and the remainder “unchanged”. A series of analyses of covariance (ANCOVAs) was conducted to compare PedsQL-MFS post-intervention mean scores across change categories (improved, unchanged, deteriorated) for the FSS, CES-D and BMI, adjusting for pre-intervention scores, age, and gender. For ROC analyses, FSS, CES-D and BMI change was dichotomized into “improved” and “not improved” and each predicted separately by changes in PedsQL-MFS Total and subscales scores. Area under the curve (AUC) values > 0.70 are generally considered to indicate acceptable responsiveness^35^. All analyses were conducted in SPSS 27.0 except for confidence intervals for Glass’s Δ (*d*) which were calculated via the MBESS package in *R*.

## Results

Table 1 reports demographic characteristics of the sample. The majority of the sample was female, reflecting the gender prevalence in the treatment center.

**Table 1:** Sample characteristics (*n* = 198).

Gender: 80.8% females
Age: *M* = 44.7 years (*SD* = 14.6)
Height: *M* = 1.63 m (*SD* = 0.1)
Weight: *M* = 118.3 kg (*SD* = 20.8)
BMI: *M* = 44.4 (*SD* = 6.1)
**Highest education level completed**
Primary school: 8.6%
Middle school (grades 6–8): 42.4%
Senior High School (grades 9–13): 41.4%
University: 7.6%
**Occupation**
Unemployed: 44.9%
Employed: 44.9%
Retired: 9.1%
Student: 1%

### Internal responsiveness

There were significant improvements in all outcome measures by the end of the 3-week body weight reduction program (see Table 2). Values for SMR were slightly higher than for effect size although they were only clearly different for BMI; only for BMI did the confidence intervals of the SMR and ES estimate not overlap. When comparing SMRs across outcome measures, BMI showed greater change than any other measure (the upper limit of the confidence interval for the SMR of the variable showing the next largest change—FSS—did not overlap with the lower limit of the confidence interval for the SMR of BMI). There was also greater change in FSS than CES-D. When comparing Glass’s delta values (*d*), BMI showed the least change, less than the PedsQL-General Fatigue subscale or FSS. There was significant overlap between the effect sizes of each fatigue measure; the PedsQL-MFS total and FSS produced similar effect size and SMR values. Within the PedsQL-MFS, effect sizes overlapped, but SMR values indicated greater change on the General Fatigue subscale than the Sleep/Rest or Cognitive Fatigue subscale.

**Table 2:** Internal Responsiveness and pre-post changes of patient reported outcomes (n = 198).

	Pre mean (SD)	Post mean (SD)	Pre-post difference value (95% CI)	Pre-post difference *p*	ES (*d*) value (95% CI)	SMR value (95%CI)
PedsQL Total	27.74 (13.13)	20.34 (11.39)	7.40 (6.1 to 8.7)	< .001	0.56 (0.36 to 0.77)	0.77 (0.62 to 0.93)
PedsQL General	10.78 (5.61)	7.09 (4.84)	3.69 (3.05 to 4.34)	< .001	0.66 (0.45 to 0.86)	0.80 (0.64 to 0.96)
PedsQL Sleep/Rest	8.78 (4.56)	6.97 (3.94)	1.80 (1.25 to 2.36)	< .001	0.39 (0.19 to 0.60)	0.46 (0.31 to 0.60)
PedsQL Cognitive	8.19 (5.14)	6.28 (4.62)	1.91 (1.36 to 2.46)	< .001	0.37 (0.17 to 0.57)	0.49 (0.34 to 0.64)
FSS	34.26 (13.61)	24.36 (12.76)	9.90 (8.23 to 11.58)	< .001	0.73 (0.52 to 0.94)	0.83 (0.67 to 0.99)
CES-D	34.85 (10.31)	30.41 (8.82)	4.44 (3.25 to 5.63)	.019	0.43 (0.23 to 0.63)	0.52 (0.37 to 0.67)
BMI	44.31 (5.95)	42.80 (5.83)	1.51 (1.42 to 1.60)	< .001	0.25 (0.05 to 0.45)	2.29 (2.02 to 2.55)

### External responsiveness

Dividing participants into groups based on minimal detectable change for FSS resulted in 150 (75.8%) “improved”, 26 (13.1%) “unchanged” and 22 (11.1%) “deteriorated”. Table 3 reports pre- and post-intervention PedsQL-MFS Total and subscale raw mean scores for each FSS change category. There was a significant effect of FSS change category on post-intervention PedsQL-MFS Total scores (*F* ^2,192^ = 10.50, *p* < 0.001). The “improved FSS” group reported significantly lower PedsQL-MFS Total scores than the “unchanged” (*Mean*_DIFF_ = 6.98 [3.02, 10.94], *p* < 0.001) and “deteriorated” groups (*Mean*_DIFF_ = 5.00 [0.74, 9.27], *p* = 0.015). There was a significant effect of FSS change group on post-intervention PedsQL-MFS General Fatigue scores, adjusting for pre-intervention General Fatigue (*F*[2,192] = 6.58, *p* = 0.002). Those who improved FSS had significantly lower General Fatigue scores than those who were “unchanged” (*Mean*_DIFF_ = 2.87 [0.97, 4.76], *p* = 0.001), although not significantly lower than the “deteriorated” group (*Mean*_DIFF_ = 1.10 [−0.96, 3.15], *p* = 0.597). The same pattern was evident for Sleep/Rest Fatigue scores, with a main effect of FSS change group (*F* [2, 192] = 5.42, *p* = 0.005) and significantly lower scores in the “improved” group than the “unchanged” group (*Mean*_DIFF_ = 1.93 [0.31, 3.55], *p* = 0.014), although the difference between “improved” and “deteriorated” groups only approached significance (Sleep/Rest Fatigue: *Mean*_DIFF_ = 1.65 [−0.10, 3.39], *p* = 0.071). Cognitive Fatigue scale post-intervention scores were significantly different between FSS change groups (*F* [2, 192] = 7.74, *p* < 0.001), being significantly lower in the “improved” group than either the “unchanged” (*Mean*_DIFF_ = 2.12 [0.44, 3.79], *p* = 0.008 ) or “deteriorated” groups (*M*_DIFF_ = 2.12 [0.44, 3.79], *p* = 0.008).

**Table 3:** Mean differences in PedsQL-MFS scores according to FSS change category.

	Pre mean (SD)	Post mean (SD)	Pre-post difference value (95% CI)	ES (d) value (95% CI)	SMR value (95%CI)
**Total fatigue: *F* [2, 192] = 10.50, *p* < .001**					
Improved	27.70 (12.83)	18.84 (10.74)	8.86 (7.43 to 10.29)	0.69 (0.45 to 0.93)	1.00 (0.80 to 1.20)
Unchanged	24.81 (14.86)	24.04 (14.55)	0.77 (−2.38 to 3.92)	0.05 (−0.49 to 0.60)	0.10 (−0.29 to 0.48)
Deteriorated	31.50 (12.64)	26.18 (8.86)	5.32 (−0.19 to 10.84)	0.42 (−0.19 to 1.02)	0.43 (−0.01 to 0.86)
**General fatigue: *F*[2,192] = 6.58, *p* = .002**					
Improved	10.49 (5.37)	6.43 (4.40)	4.05 (3.36 to 4.74)	0.75 (0.51 to 1.00)	0.95 (0.76 to 1.14)
Unchanged	10.50 (6.51)	9.31 (6.88)	1.19 (−0.34 to 2.72)	0.18 (−0.36 to 0.73)	0.32 (−0.08 to 0.71)
Deteriorated	13.09 (5.84)	8.91 (3.64)	4.18 (1.25 to 7.11)	0.72 (0.08 to 1.34)	0.63 (0.17 to 1.09)
**Sleep/rest fatigue: *F* [2, 192] = 5.42, *p* = .005**					
Improved	8.78 (4.38)	6.54 (3.78)	2.24 (1.62 to 2.86)	0.51 (0.28 to 0.74)	0.58 (0.41 to 0.76)
Unchanged	7.92 (5.43)	8.04 (4.65)	−0.12 (−1.50 to 1.27)	0.02 (−0.52 to 0.57)	−0.03 (−0.42 to 0.35)
Deteriorated	9.77 (4.73)	8.68 (3.60)	1.09 (−0.97 to 3.15)	0.23 (−0.37 to 0.82)	0.43 (−0.01 to 0.86)
**Cognitive fatigue: *F* [2, 192] = 7.74, *p* < .001**					
Improved	8.43 (5.29)	5.87 (4.66)	2.57 (1.97 to 3.16)	0.48 (0.25 to 0.72)	0.70 (0.52 to 0.87)
Unchanged	6.39 (4.69)	6.69 (4.86)	−0.31 (−1.54 to 0.92)	−0.06 (−0.48 to 0.61)	−0.10 (−0.49 to 0.29)
Deteriorated	8.64 (4.36)	8.59 (3.35)	0.05 (−2.04 to 2.13)	0.01 (−0.58 to 0.60)	0.01 (−0.40 to 0.42)

Using CES−D as an anchor with a RCI of 13.53 to divide response categories, only 2 participants evidenced reliable deterioration in depression symptoms, leaving 33 (16.7%) “improved” and 163 (82.3%) “unchanged”. Table 4 reports pre- and post-intervention means in PedsQL-MFS scale scores—and for comparison, the FSS—separately for those improved and those “not improved” (combined unchanged or deteriorated) on the CES-D and the result of the ANCOVA for the effect of CESD group on postintervention scores adjusting for preintervention values on those scores. For all PedsQL-MFS scales the SRM for reduction in fatigue symptoms in the “improved” group were larger than the small to moderate effect size reductions in the “not improved” group (confidence intervals do not overlap). For each PedsQL-MFS scale, post-intervention scores were significantly lower in the “improved” CES-D change group than the “not improved” group when adjusting for pre-intervention values. By comparison, the corresponding difference in FSS post-intervention scores approached but did not reach statistical significance.

**Table 4:** Mean differences in PedsQL-MFS scores according to CESD change category.

	Pre mean (SD)	Post mean (SD)	Pre-post difference value (95% CI)	ES (*d*) value (95% CI)	SMR value (95%CI)
**Total fatigue: *F*(1, 193) = 26.59, *p* < .001**					
Improved	35.85 (13.60)	19.33 (11.27)	16.52 (13.44 to 19.60)	1.21 (0.64 to 1.77)	1.90 (1.32 to 2.47)
Not improved	26.12 (12.45)	20.54 (11.44)	5.58 (4.25 to 6.91)	0.45 (0.23 to 0.67)	0.64 (0.48 to 0.81)
**General fatigue: *F*(1, 193) = 14.88, *p* < .001**					
Improved	14.76 (5.45)	7.06 (4.96)	7.70 (6.08 to 9.32)	1.41 (0.81 to 2.00)	1.68 (1.14 to 2.21)
Not improved	9.98 (5.31)	7.09 (4.83)	2.89 (2.25 to 3.53)	0.54 (0.32 to 0.76)	0.69 (0.52 to 0.86)
**Sleep/rest fatigue: *F*(1, 193) = 14.07, *p* < .001**					
Improved	11.30 (4.97)	6.42 (4.04)	4.88 (3.59 to 6.17)	0.98 (0.44 to 1.51)	1.34 (0.86 to 1.81)
Not improved	8.27 (4.32)	7.08 (3.93)	1.19 (0.61 to 1.76)	0.28 (0.06 to 0.49)	0.32 (0.16 to 0.47)
**Cognitive fatigue: *F*(1, 193) = 8.46, *p* = .004**					
Improved	9.79 (6.13)	5.85 (4.29)	3.93 (2.24 to 5.64)	0.64 (0.13 to 1.14)	0.82 (0.42 to 1.21)
Not improved	7.87 (4.88)	6.36 (4.69)	1.50 (0.95 to 2.05)	0.31 (0.09 to 0.53)	0.42 (0.26 to 0.58)
**FSS: *F*(1, 193) = 2.75, *p* = .099**					
Improved	39.93 (12.58)	24.85 (11.66)	15.09 (10.36 to 19.83)	1.20 (0.63 to 1.76)	1.13 (0.68 to 1.56)
Not Improved	33.13 (13.56)	24.26 (13.00)	8.87 (7.11 to 10.62)	0.70 (0.47 to 0.93)	0.78 (0.60 to 0.95)

Using BMI change as an anchor, 27 participants (13.6%) made a 5% or greater reduction in BMI (“improved”), with 171 (86.4%) achieving less BMI reduction (“unchanged”). There was no significant increase in weight. Table 5 reports the mean pre- and post-intervention PedsQL-MFS—and for comparison, FSS—scores and within-group effect sizes according to BMI change status group. There were no significant differences in PedsQL-MFS scale scores or FSS scores between participants who had achieved 5% change in BMI and those who had not. Effect sizes for changes in PedsQL-MFS fatigue symptoms were similar (confidence intervals overlapped) for those both with and without clinically significant weight loss.

**Table 5:** Mean differences in PedsQL-MFS scores according to BMI change category where significant BMI change ≥ 5%.

	Pre mean (SD)	Post mean (SD)	Pre-post difference value (95% CI)	ES (*d*) value (95% CI)	SMR value (95%CI)
**Total fatigue: *F*(1,193) = 0.28, *p* = .596**					
Improved	24.04 (13.13)	17.19 (10.95)	6.85 (3.27 to 10.44)	0.52 (−0.03 to 1.07)	0.76 (0.32 to 1.18)
Unchanged	28.33 (13.07)	20.84 (11.41)	7.49 (6.03 to 8.95)	0.57 (0.35 to 0.79)	0.78 (0.60 to 0.95)
**General fatigue: *F*(1,193) = 0.04, *p* = .839**					
Improved	10.15 (5.99)	6.07 (5.33)	4.07 (2.31 to 5.84)	0.68 (0.11 t0 1.24)	0.91 (0.45 to 1.36)
Unchanged	10.88 (5.56)	7.25 (4.75)	3.63 (2.93 to 4.33)	0.65 (0.43 to 0.88)	0.78 (0.61 to 0.95)
**Sleep/rest fatigue: *F*(1,193) = 0.68, *p* = .412**					
Improved	7.78 (4.53)	6.56 (4.18)	1.22 (−0.72 to 3.17)	0.27 (−0.27 to 0.81)	0.25 (−0.14 to 0.63)
Unchanged	8.94 (4.56)	7.04 (3.91)	1.89 (1.32 to 2.47)	0.41 (0.20 to 0.63)	0.50 (0.34 to 0.66)
**Cognitive fatigue: *F*(1,193) = 0.17, *p* = .683**					
Improved	6.11 (4.99)	4.56 (3.92)	1.56 (0.50 to 2.61)	0.31 (−0.23 to 0.85)	0.58 (0.17 to 0.99)
Unchanged	8.52 (5.11)	6.55 (4.67)	1.96 (1.35 to 2.58)	0.38 (0.17 to 0.60)	0.48 (0.32 to 0.64)
**FSS: *F*(1,193) = 0.03, *p* = .875**					
Improved	34.70 (14.34)	22.37 (12.95)	12.33 (7.01 to 17.66)	0.86 (0.27 to 1.43)	0.92 (0.46 to 1.36)
Unchanged	34.19 (13.54)	24.67 (12.74)	9.52 (7.76 to 11.28)	0.70 (0.47 to 0.92)	0.82 (0.64 to 0.99)

### ROC analyses

Table 6 reported area under the curve (AUC) values for ROC models using PedsQL-MFS scales to predict treatment response as defined by FSS, CES-D. Responsiveness varied depending on the anchor. Changes in PedsQL-MFS Total, General Fatigue and Sleep/Rest Fatigue subscales were somewhat more predictive of improvement in depressive symptoms (CES-D) than fatigue severity improvement as measured by FSS. Changes in PedsQL-MFS scores were not predictive of whether participants achieved clinically significant improvement in BMI.

**Table 6:** AUC values predicting improvement in FSS and CES-D from change in PedsQL-MFS total and subscales.

	FSS	CES-D	BMI
PedsQL-MFS total change	0.70 [0.61, 0.80]**	0.84 [0.77, 0.90]**	0.51 [0.39, 0.63]
General fatigue change	0.61 [0.51, 0.71]*	0.79 [0.71, 0.87]**	0.54 [0.42, 0.67]
Sleep/rest fatigue change	0.66 [0.57, 0.75]*	0.78 [0.70, 0.86]**	0.45 [0.34, 0.57]
Cognitive fatigue change	0.70 [0.61, 0.79]**	0.65 [0.54, 0.76]*	0.48 [0.37, 0.59]

*p < .05.

**p < .001.

### Correlations between change variables

Table 7 reports bivariate correlations between change scores (i.e., the difference between pre-intervention and post-intervention score) with 95% bias-corrected and accelerated confidence intervals for each variable. There were moderate correlations between changes in each of the PedsQL-MFS subscales. Changes in FSS scores were only weakly correlated with changes in the PedsQL-MFS subscales. Changes in depressive symptoms were more strongly correlated with changes in the PedsQL-MFS scales than changes in the FSS. None of the changes in self-reported psychological symptoms were significantly correlated with changes in percentage of BMI.

**Table 7:** Correlations between change scores.

	PedsQL-MFS general fatigue	PedsQL-MFS sleep/rest fatigue	PedsQL-MFS cognitive fatigue	FSS	CESD	BMI
PedsQL-MFS Total Fatigue	0.80 [0.73, 0.85]***	0.79 [0.72, 0.84]***	0.71 [0.62, 0.79]***	0.27 [0.10, 0.44]***	0.51 [0.40, 0.62]***	0.04 [−0.11, 0.20]
PedsQL-MFS General Fatigue	–	0.45 [0.32, 0.58]***	0.31 [0.15, 0.47]***	0.20 [0.01, 0.38]**	0.40 [0.28, 0.53]***	0.04 [−0.11, 0.18]
PedsQL-MFS Sleep/Rest Fatigue	–	–	0.38 [0.22, 0.51]***	0.22 [0.03, 0.39]**	0.45 [0.32, 0.57]***	0.03 [−0.12, 0.19]
PedsQL-MFS Cognitive Fatigue			–	0.20 [0.06, 0.34]**	0.33 [0.16, 0.46]***	0.02 [−0.10, 0.15]
FSS				–	0.17 [−0.02, 0.37]*	0.12 [−0.03, 0.26]
CESD					–	0.04 [−0.11, 0.20]

*p < .05.

**p < .01.

***p < .001.

## Discussion

This is the first investigation of the responsiveness of the Italian version of the PedsQL-MFS to changes in an adult inpatient body weight reduction program. The PedsQL-MFS demonstrated internal responsiveness through medium to large effect size changes in response to a body weight reduction with previously demonstrated effectiveness^24^. The PedsQL-MFS subscales were differentially responsive with the General Fatigue scale showing significantly larger changes in response to the program than the Sleep/Rest or Cognitive Fatigue subscales. The General Fatigue subscale demonstrated similar responsiveness as the FSS. The PedsQL-MFS generally demonstrated external responsiveness when using self-report measures as anchors. In comparing those who self-reported improvement in depressive symptoms with those who did not, those whose depression improved reported lower postintervention PedsQL-MFS scores on the Total and all subscales. Post-intervention PedsQL-MFS Total and Cognitive Fatigue scores were also significantly lower for those who reported improvement on the FSS than those whose scores were unchanged or worsened. The General Fatigue and Sleep/Rest Fatigue subscales were both significantly lower among those who had improved on the FSS than those whose scores were unchanged, but unexpectedly were not significantly different to those whose FSS scores deteriorated. Only a relatively small proportion of the sample reported worsening of fatigue symptoms on the FSS and there was considerable variability in the amount of change in General Fatigue scale scores among this group. Nevertheless, it is possible that the General Fatigue and Sleep/Rest subscale items are less sensitive to deterioration in fatigue than improvement. On the other hand, PedsQL-MFS scale scores were not significantly different between those who had reduced their BMI by 5% or more and those who had not. The current data suggest the PedsQL-MFS is sensitive to improvements in psychological functioning associated with weight loss, rather than weight loss per se.

ROC analyses found that the PedsQL-MFS Total and General Fatigue subscale predicted reliable improvement in CES-D scores with greater accuracy than they predicted improvement in FSS scores. These findings could indicate either that the PedsQL-MFS may be more sensitive to improvements in depressive symptoms than fatigue, or that the FSS does not capture fatigue as fully as the PedsQL-MFS scales.

The strong association between improvements in depression and fatigue in the present study continues a consistent pattern in the literature. More than two thirds of people with depression report fatigue as a symptom^38^. Depression is a major predictor of fatigue in rheumatoid arthritis^39^ and paediatric fatigue across a range of chronic diseases^40^. Depression and fatigue have been strongly associated in obese samples^9^. Multiple shared aetiologies between depression and fatigue have been proposed including systemic inflammation, mitochondrial dysfunction, oxidative stress, autoimmune abnormalities, hypothalamic-pituitary axis dysfunction, and structural and functional brain changes^41,42^. Strong associations between fatigue and depression have been found previously using the PedsQL-MFS^16^ and the FSS (e.g.,^43^) although the original validation study emphasized its independence from depression^31^. Depression and fatigue are overlapping but distinct aspects of quality of life which are adversely affected by obesity, responsive to treatment, and important outcomes to consider in evaluating obesity intervention.

The relative independence of psychological changes from physical changes in the present study is also relatively common in the literature. In rheumatoid arthritis, psychosocial predictors have been found to be better predictors of fatigue than physical factors; in fact, lower disease activity was associated with increased fatigue^39^. Fatigue has been found to be relatively unrelated to physical disease severity in rheumatoid arthritis^44^, paediatric immune thrombocytopenia^45^, objective sleep in children with epilepsy^46^ and TBI severity^47^. In people with obesity, correlations between improvements in body weight reduction and fatigue have been relatively weak. Rigamonti and colleagues^48^ found similar improvements in fatigue among those with and without metabolic disorder. Those with metabolic disorder retained higher body weight after the intervention than those without, suggesting that weight reduction was less important for improvements in fatigue than psychological changes. In a cross-sectional study,^49^ found weak correlations between fatigue and each of BMI, total body fat and visceral fat. Even though it may be primarily a psychological construct, fatigue should not be minimized as an important outcome of weight reduction interventions, as it is among the most severe and distressing symptoms reported by patients across a range of problems including cancer, chronic fatigue syndrome, multiple sclerosis, Parkinson’s disease and Major Depressive Disorder^50^.

That the General Fatigue subscale appeared to show larger changes than Sleep/Rest or Cognitive Fatigue subscales is difficult to interpret in the absence of other measures of sleep or cognitive fatigue. These differences might reflect the nature of the intervention rather than the PedsQL-MFS. Sheng and colleagues^51^ reported improvements in physical functioning and fatigue in obese breast cancer-surviving women in response to a remotely delivered weight loss program, but not sleep. Moderate-intensity aerobic and resistance exercise training was found to significantly reduce fatigue in obese adults with obstructive sleep apnea but improvements in daytime sleepiness were not significantly better than those achieved in the low-intensity stretching control group^52^. There is evidence that although there is a dose–response relationship between weight loss and excessive daytime sleepiness, it is non-linear, with diminishing improvements in sleepiness with increasing weight loss^53^. Studies that employ the PedsQL-MFS and other measures of excessive daytime sleepiness and nighttime sleep quality would be needed to disentangle the effect of the intervention from the psychometric properties of the PedsQL-MFS.

It is similarly difficult to know whether smaller changes in the Cognitive Fatigue subscale reflect lower responsiveness in the PedsQL-MFS or whether this reflects a differential treatment effect. There is little information about differential fatigue profiles following weight reduction programs in the literature, although one study found that physical fatigue showed significantly greater improvement than mental fatigue six months after gastric bypass surgery^54^. Again, studies that employ the PedsQL-MFS in conjunction with other measures of mental fatigue would be needed to evaluate the fully evaluate the responsiveness of the Cognitive Fatigue subscale.

Although the PedsQL-MFS was responsive to improvement in FSS and CES-D scores, its subscales were not as responsive to deterioration as detected by the FSS in the present study; in other words, reductions in scores between pre- and post-intervention were not significantly lower among those who showed reliable deterioration compared to those who showed reliable improvement on the FSS. Despite previous concerns over its floor effects^16^, floor and ceiling effects were within acceptable limits and would not account for the PedsQL-MFS’s insensitivity to deterioration.

The present findings are relevant to researchers looking to select measures to evaluate weight reduction programs. The FSS and PedsQL-MFS were similarly responsive to the intervention, but changes in each were only weakly correlated with changes in the other. The PedsQL-MFS scale scores reflected the differences between those who had, and had not, achieved reliably improved depression symptoms, whereas the FSS did not; those who had not made significant improvement in depressive symptoms still demonstrated a reasonably large reduction in fatigue symptoms according to the FSS. The strength of the present PedsQL-MFS change – FSS change correlations are somewhat weaker than previously found with the FSS and the Profile Of Moods Scale Fatigue scale^25^, although still within the range of overlapping confidence intervals. The PedsQL-MFS was concertedly designed to be multidimensional and explicitly represent the effects of cognitive fatigue and sleep/rest fatigue, whereas the FSS was designed to be a unidimensional measure of the overall impact of fatigue across all domains of functioning. The findings suggest that researchers interested in a more nuanced understanding of how fatigue responds to intervention can obtain distinct information from the PedsQL-MFS subscales. The relatively low overlap between the FSS and the PedsQL-MFS General Fatigue subscale is surprising and not readily explainable. In studies of the FSS in other populations and other translations, it has generally correlated highly with other general measures of fatigue (e.g.,^55,56^). On the other hand, it has been found less sensitive to detecting the memory deficits of cognitive fatigue in traumatic brain injury than the Multidimensional Assessment of Fatigue (^57^). Furthermore, as a unidimensional measure of general fatigue, Rasch analysis of the original version of the FSS has revealed certain limitations: some of the response categories are rarely used, items 1 and 2 don’t appear to fit with the rest of the scale, and the FSS only covers a limited range of the latent fatigue construct (^58,59^). The FSS has been reported to be less precise in measuring fatigue at very low or very high levels^55^. It is difficult to compare the PedsQL-MFS however because to our knowledge, it has not been subjected to similar Rasch analysis. Future studies might compare the PedsQL-MFS and FSS with other measures of fatigue and related constructs such as sleepiness, vitality and focus to better characterize which aspects of fatigue they each represent. For now, the Italian versions of each of these instruments do not overlap sufficiently that one be replaced with the other, so we recommend they both be administered in program evaluations.

The strengths of this study include having a large clinical sample of adults with diverse age and education level, a program known to be effective to measure the responsiveness of the PedsQL-MFS, and a range of measures including a measure of fatigue with demonstrated responsiveness as an external anchor. The ability of a measure to detect clinically important deterioration is arguably more important in making clinical decisions about the treatment of an individual than its sensitivity to improvement. Given that the PedsQL-MFS Total score appeared to display adequate responsiveness, clinicians are encouraged to monitor this score rather than the subscale totals to assess deleterious responses to interventions.

The main limitation of the study is that due to the relative brevity of the intervention for the severity of obesity, the groups of “responders” were uneven, such that the majority did not evidence change. Nevertheless, there was sufficient power to demonstrate sensitivity to improvement when psychological measures served as external anchors. There was no reliable deterioration in depression symptoms during the program which suggests that perhaps the positive effects on mental health of engaging with a multidisciplinary team and the group cohesion of undergoing the program with others experiencing similar challenges buffered against any worsening of fatigue symptoms. This would be consistent with some authors’ view that fatigue primarily reflects psychological adjustment to obesity rather than physiological status^19^. For the purposes of the present study, a program with ubiquitously positive to benign effects on mental health may not be well placed to detect reliable deterioration in fatigue. The lack of no-treatment control group is thus a further limitation of the study, which might have made detection of deterioration more feasible.

In conclusion, the PedsQL-MFS can detect improvements in fatigue and depression symptoms. Future research should include a no treatment (or delayed treatment) control group to explore the responsiveness of the PedsQL-MFS when deterioration in fatigue is more likely to occur. For now, the Italian version of the PedsQL-MFS can be recommended as an outcome measure for evaluating improvement from inpatient multidisciplinary body weight reduction programs.

## Data Availability

Raw data will be available upon a reasonable request to the corresponding author and will be uploaded on zenodo.org when we will receive confirmation of acceptance of the ms.
